# Tetramethylpyrazine Protects Endothelial Injury and Antithrombosis via Antioxidant and Antiapoptosis in HUVECs and Zebrafish

**DOI:** 10.1155/2022/2232365

**Published:** 2022-07-18

**Authors:** Yafang Zhang, Cheng Ma, Linfeng He, Li Liao, Chaocheng Guo, Cheng Wang, Lihong Gong, Honglin Zhou, Ke Fu, Cheng Peng, Yunxia Li

**Affiliations:** ^1^State Key Laboratory of Southwestern Chinese Medicine Resources, Chengdu 611137, China; ^2^School of Pharmacy, Chengdu University of Traditional Chinese Medicine, Chengdu 611137, China; ^3^Key Laboratory of Standardization for Chinese Herbal Medicine, Ministry of Education, Chengdu 611137, China

## Abstract

Chuanxiong Rhizoma, the dried rhizome of *Ligusticum chuanxiong* Hort., is a commonly used drug for promoting blood circulation and dissipating congestion. Tetramethylpyrazine (TMP), the main active ingredient of *Ligusticum chuanxiong*, has significant antioxidant, anti-inflammatory, and vascular protective effects. However, the protective properties and underlying mechanisms of TMP against endothelial injury-induced insufficient angiogenesis and thrombosis have not been elucidated. Therefore, we aimed to explore the protective effects of TMP on endothelial injury and its antithrombotic effects and study the mechanism. *In vitro* experiments showed that TMP could alleviate hydrogen peroxide– (H_2_O_2_–) induced endothelial injury of human umbilical vein endothelial cells (HUVECs) and the protective mechanism might be related to the regulation of MAPK signaling pathway, and its antioxidative and antiapoptotic effects. *In vivo* experiments showed that TMP restored PTK787-induced damage to intersegmental vessels (ISVs) in *Tg(fli-1: EGFP)y1* transgenic (Flik) zebrafish larvae. Similarly, adrenalin hydrochloride– (AH–) induced reactive oxygen species (ROS) production and thrombosis in AB strain zebrafish were inhibited by TMP. RT-qPCR assay proved that TMP could inhibit the expression of fga, fgb, fgg, f7, and von Willebrand factor (vWF) mRNA to exert an antithrombotic effect. Our findings suggest that TMP can contribute to endothelial injury protection and antithrombosis by modulating MAPK signaling and attenuating oxidative stress and antiapoptosis.

## 1. Introduction

Thrombosis often causes cardiovascular diseases such as coronary heart disease, atherosclerosis, ischemic stroke, and myocardial infarction, which has a profound impact on people's quality of life [1]. At present, the most commonly used antithrombotic drugs are warfarin, aspirin, and heparin, but they are often accompanied by some side effects such as bleeding and drug resistance [2]. Therefore, it is necessary to study the molecular mechanism of thrombosis and explore alternative antithrombotic therapies.

It is generally accepted that oxidative stress and apoptosis are major factors associated with endothelial injury and thrombosis in cardiovascular diseases. Mitochondria are the main producers of reactive oxygen species (ROS), and the imbalance between the overproduction of mitochondrial ROS (mROS) and the antioxidant defense system leads to oxidative stress, which is a key initial step in the pathogenesis of many vascular diseases [3]. And oxidative damage is thought to lead to the initiation of endothelial injury and coagulation cascade [4]. On the other hand, excess ROS can lead to increased permeability of mitochondrial membrane, and various proteins and proteases related to apoptosis are released into the cytoplasm, leading to apoptosis and ultimately promoting thrombus formation. Therefore, studying oxidative stress and apoptosis in endothelial cells may provide a better understanding of the pathogenesis of thrombosis.

Chuanxiong Rhizoma, the dried rhizome of *Ligusticum chuanxiong* Hort., is one of the commonly used drugs for promoting blood circulation and removing blood stasis, and has protective effects on cardiovascular and cerebrovascular diseases [5]. Tetramethylpyrazine (TMP), as one of the important alkaloids extracted from *Ligusticum chuanxiong*, has pharmacological activities such as anti-inflammatory, antioxidation, antiplatelet aggregation, antiapoptosis, and improving microcirculation [6–9]. It is known that the MAPK pathway can be activated by hydrogen peroxide (H_2_O_2_), thereby regulating various cellular activities, including proliferation, differentiation, survival, and death. However, whether TMP exerts its antithrombotic effect by protecting endothelial injury through the MAPK pathway has not been thoroughly studied. Therefore, in this study, human umbilical vein endothelial cells (HUVECs) were used to establish H_2_O_2_-induced a H_2_O_2_-induced oxidative damage model, and zebrafish larvae were used to establish thrombosis and angiogenesis deficiency models to evaluate the vascular endothelial protection and antithrombotic activities of TMP and lay a molecular basis and therapeutic potential for TMP to prevent cardiovascular diseases.

## 2. Materials and Methods

### 2.1. Drug and Reagents

**Figure 1:**
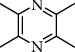
The chemical structure of tetramethylpyrazine (TMP).

TMP (purity≥99.95%, the chemical structure of TMP shown in Figure 1) was purchased from the Must Bio-Technology Company, China. 1640 medium and fetal bovine serum (FBS) were purchased from Gibco (Australia). Trypsin (1 : 250) was purchased from BioFroxx (Guangzhou, China). HUVECs were obtained from the school of pharmacy, Chengdu University of Traditional Chinese Medicine (Sichuan, China). 3-(4,5-dimethyl-thiazol-2-yl)-2,5-diphenyltetrazolium bromide (MTT) was from Biosharp (Beijing, China). Malondialdehyde (MDA) and superoxide dismutase (SOD) commercial kits were obtained from Elabscience Biotechnology Co., Ltd (Wuhan, China). Reactive oxygen species assay kit was purchased from Yeasen (Shanghai, China). BCA protein assay kit, PMSF, and Ripa lysis buffer were purchased from Beyotime (Jiangsu, China). Cell Total RNA Isolation Kit was purchased from Fuji Biotechnology Co., Ltd (Chengdu, China), 5 × All-In-one MasterMix and Eva Green 2 × RT-qPCR MasterMix-Low RoX were purchased from ABclonal Technology Co., Ltd. (Wuhan, China). Specific rabbit polyclonal antibodies to p38, MEK1/2, and ERK1/2 were purchased from Bimake (Shanghai, China); phospho-p38, phospho-MEK1/2, phospho-ERK1/2, and *β*-actin (as a loading control) were purchased from Affinity Biosciences. PTK787 CAS: 212141-51-0 was purchased from MedChemExpress (New Jersey, USA). Adrenalin hydrochloride injection was purchased from Dikang Changjiang Pharmaceutical Co., Ltd. Other chemicals and reagents used in this study were obtained from Kelong Chemical Reagent Factory (Chengdu, China).

### 2.2. Cell Culture

HUVECs were cultured in 1640 medium supplemented with 100 U/mL penicillin, 100 U/mL streptomycin, and 10% FBS. All cells incubated at 37 °C with 95% humidity and 5% CO_2_.

### 2.3. Determination of TMP Concentration

MTT assay was used to study the cytotoxicity of TMP on HUVECs. Cells were seeded at a density of 8 × 10^3^ in sterile 96-well plates and incubated at 37 °C for 24 h, using 10% FBS-1640 medium, 100 *μ*L/well. Cells were treated with various concentrations of TMP (5-120 *μ*g/mL) dissolved in DMSO, and medium containing 1% FBS was used. The control group only cultured 1640 medium containing 1% FBS in 5% CO_2_ incubator at 37 °C for 24 h. Then, add 20 *μ*L MTT solution to each well, and incubate at 37 °C for 4 h in the dark. After that, the MTT solution was discarded. Add 150 *μ*L DMSO solution to each well at room temperature to lyse cells and mix for 5 min. Finally, the absorbance was determined at 570 nm with a BIO-RAD microplate reader (Benchmark Plus, USA).

### 2.4. Oxidative Stress Injury Model

Cells were seeded in a sterile 96-well plates at a cell density of 8 × 10^3^ for 24 h. HUVECs were exposed to H_2_O_2_ (50-800 *μ*M) for 24 h, and cell viability was detected by MTT as described in Section 2.3.

### 2.5. Cell Viability Test

Cells were seeded in sterile 96-well plates at a cell density of 8 × 10^3^ for 24 h. HUVECs were exposed to TMP (10, 20, and 40 *μ*g/mL) and H_2_O_2_ (100 *μ*M) for 24 h, and cell viability was detected by MTT as described in Section 2.3.

### 2.6. Wound Healing Assay

HUVECs were seeded in 6-well plates and grown to about 80% fusion rate. Scratch with 200 *μ*L sterile plastic pipette tip, and wash with PBS to remove non-adherent and damaged cells. The model group was treated with H_2_O_2_ (100 *μ*M), and the administration groups were treated with different concentrations of TMP (10, 20, and 40 *μ*g/mL) and H_2_O_2_ (100 *μ*M) and then incubated with basal medium containing 1% FBS for 24 h. Image of the wounds were taken in five random fields (×100) by an inverted microscope (Leica DMI3000 B, Germany) at 0 h and 24 h. The wound healing areas were calculated by Image J, and the formula of wound healing ability is as follows: Wound closure% = (wound areas on 0 h − wound areas on 12 h)/wound areas on 0 h × 100%.

### 2.7. Measurement of mROS

HUVECs were seeded in 6-well plates and grown for 24 h. Then, the model group was treated with H_2_O_2_ (100 *μ*M), and the TMP groups was treated with different concentrations of TMP (10, 20, and 40 *μ*g/mL) and H_2_O_2_ (100 *μ*M) for 24 h. Cells were washed with PBS and incubated with 5 *μ*M MitoSOX Red was dyed at 37 °C in the dark for 0.5 h. Images were taken under a fluorescence microscope (Leica DMI3000 B, Germany). The fluorescence density of each group was analyzed by Image J software.

### 2.8. MDA and SOD Analysis

HUVECs were seeded in 6-well plates and grown for 24 h. Then, the model group was treated with H_2_O_2_ (100 *μ*M), and the TMP groups was treated with different concentrations of TMP (10, 20, and 40 *μ*g/mL) and H_2_O_2_ (100 *μ*M) for 24 h. The cells in each group were digested and collected with PBS, and the cell homogenate was obtained by ultrasonic crushing. According to the manufacturer instructions, the absorbance of MDA and SOD was measured at 550 and 532 nm to detect their activity in the cell homogenate.

### 2.9. Apoptosis Assay

HUVECs were seeded in 6-well plates and grown for 24 h. Then, the model group was treated with H_2_O_2_ (100 *μ*M), and the administration groups was treated with different concentrations of TMP (10, 20, and 40 *μ*g/mL) and H_2_O_2_ (100 *μ*M) for 24 h. Then, the cells were collected by centrifugation at 1000 × *g* for 10 min and washed once with precooled PBS. According to the introduction of the Annexin V-FITC/PI Apoptosis Detection Kit, cells were resuspended in 195 *μ*L binding buffer and incubated with 5 *μ*L Annexin V-FITC and 10 *μ*L PI for 10-20 min at room temperature in the dark. Apoptotic cells were detected by using a FACSCalibur flow cytometer (BD Biosciences, San Jose, CA).

### 2.10. Western Blot Assay

HUVECs were seeded in 6-well plates and grown for 24 h. Then, the model group was treated with H_2_O_2_ (100 *μ*M), and the TMP groups was treated with different concentrations of TMP (10, 20, and 40 *μ*g/mL) and H_2_O_2_ (100 *μ*M) for 24 h. Cell lysates were centrifuged at 12,000 × *g* for 15 min at 4 °C. The protein concentration of each sample supernatant was measured using BCA protein analysis kit. Add protein loading buffer (total protein : loading buffer = 4 : 1) and heat at 100 °C for 10 min. Each group of proteins was equally loaded on 10% SDS-PAGE and transferred to PVDF membrane, which were blocked with 5% skim milk for 2 h at room temperature. Incubate overnight at 4 °C with primary antibodies (GAPDH, p38 MAPK, MEK1/2, ERK1/2, phospho-p38 MAPK, phospho-MEK1/2, phospho-ERK1/2, Bcl-2, Bax, caspase-3, cleaved-caspase-3 (activated state of caspase-3), cytochrome c at a dilution of 1 : 1000. Subsequently, after washing three times with TBST, membranes were incubated with horseradish peroxidase– (HRP–) conjugated goat anti-rabbit IgG (1 : 5000) for 2 h at room temperature. Protein bands were detected with ECL kit and quantified with Image J software.

### 2.11. Animal Maintenance

Both the AB strain zebrafish and *Tg(fli-1: EGFP)y1* transgenic (Flik) zebrafish were provided by China Zebrafish Resource Center (Wuhan, China) and maintained according to the Zebrafish Handbook [10]. Adult zebrafish were kept in a breeding system of 28 ± 0.5°C and 14 h/10 h light/dark cycle. Adult male zebrafish and female zebrafish (3-12 months old) were naturally mated and spawned in a 2 : 1 ratio. Collect embryos, transfer to petri dishes with fresh embryo medium, and culture at 28.5 °C for further experiments. All zebrafish assays were approved by the Ethics Committee of the Biology Institute of Sichuan Academy of Science.

### 2.12. Experimental Procedures for Zebrafish Assays

#### 2.12.1. Evaluation of the Proangiogenic Activity of TMP on PTK787-Induced ISV Insufficiency in Flik Zebrafish Larvae

The 24 hpf (hours post fertilization) Flik zebrafish embryos were placed in a 24-well microplate (10 larvae per well). The model group was treated with PTK787 (0.2 *μ*g/mL), and the drug groups were treated with different concentrations of TMP (10, 20, and 40 *μ*g/mL) and PTK787 (0.2 *μ*g/mL) for 24 h. Zebrafish larvae were placed laterally on glass slides, and the intersegmental vessels (ISVs) of Flik zebrafish were imaged by fluorescence inverted microscope. Intact vessels were defined as ISVs that connected the dorsal aorta to the posterior cardinal vein and elongated to the dorsal longitudinal anastomotic vessels. The ISVs index is calculated as follows: ISVs index = number of intact vessels × 1 + number of defective vessels × 0.5.

#### 2.12.2. Evaluation of the Antithrombotic Activity of TMP in AB Strain Zebrafish Larvae

The 5 dpf (days post fertilization) AB zebrafish embryos were placed in a 6-well microplate (30 larvae per well). The model group was treated with adrenalin hydrochloride (AH) (45 *μ*M), and the drug groups was treated with different concentrations of TMP (10, 20, and 40 *μ*g/mL) and AH (45 *μ*M) at 28 °C for 16 h. Then, the larvae were stained with o-anisidine at 28 °C for 30 min, and the larvae were quickly washed with DMSO three times. The caudal vein thrombosis of zebrafish larvae was observed and photographed under microscope (Leica Microsystems, Germany) and finally analyzed by Image J software. The antithrombotic effect of TMP is evaluated as follows:

(1) $$\begin{array}{c} \text{Therapeutic efficacy}\left(\%\right)=\frac{\left[\text{S}\left(\text{drug}\right)-\text{S}\left(\text{model}\right)\right]}{\left[\text{S}\left(\text{control}\right)-\text{S}\left(\text{model}\right)\right]}\times 100\%. \end{array}$$

### 2.13. ROS Analysis

Zebrafish larvae were grouped as described in Section 2.12.2. After treatment, zebrafish were washed 3 times and incubated with 25 ng/mL DCFH-DA solution for 30 min at 28.5 °C in the dark. Afterwards, the embryos were rinsed with fresh medium and photographed under a fluorescence microscope. Finally, the fluorescence intensity of zebrafish larvae was analyzed by Image J software.

### 2.14. Total RNA Extraction, Reverse Transcription, and RT-qPCR Analysis

AB zebrafish larvae were grouped and administered as described in Section 2.12.2. Total RNA was extracted using Total Animal RNA Isolation kit (FOREGENE), and the RNA concentration was measured at 260/280 nm by detecting OD. Then, the RNA was converted to single-strand cDNA with a cDNA Synthesis System for RT-PCR (ABclonal). After that, RT-PCR was performed using an Applied Biosystems 7500HT Sequence Detection System. The RT-qPCR reaction conditions were set as follows: 95 °C for 3 min, 40 cycles of 95 °C for 5 s, and 60 °C for 30 s. The expression of f7, fga, fgb, fgg, and von Willebrand factor (vWF) mRNA was analyzed according to the manufacturer protocol. GAPDH was used as an internal reference. All primer sequences (TsingKe) used are listed in Table 1.

### 2.15. Ethics Statement

Studies on zebrafish were conducted following the Experimental Animal Ethical Committee of Chengdu University of Traditional Chinese Medicine.

### 2.16. Data Analysis

All the values were presented as Mean ± S.D. The differences between groups were analyzed using Student's *t*-test when there were only two groups or assessed by one-way ANOVA when there were more than two groups. All statistical analyses were performed using GraphPad Prism 8.0 (GraphPad, San Diego, CA, USA). A two-tailed value of *P* < 0.05 was considered statistically significant.

## 3. Results

### 3.1. TMP Promoted Cell Proliferation and Inhibited H_2_O_2_-Induced Injury in HUVECs

**Figure 2:**
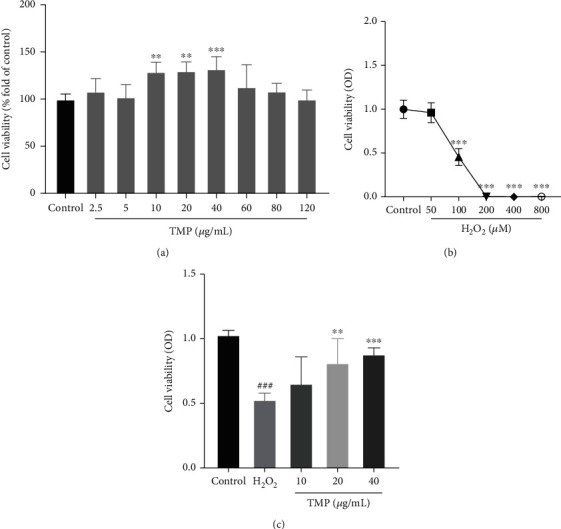
Detection of cell viability by MTT assay. (a) The effect of TMP on the viability of HUVECs. (b) The effect of H_2_O_2_ on the viability of HUVECs. (c) The viability of H_2_O_2_-induced injury following treatment with TMP at different concentrations. Values are presented as means ± S.D. (*n* = 3). #*P* < 0.05, ##*P* < 0.01, ###*P* < 0.001 vs. control group; ∗*P* < 0.05, ∗∗*P* < 0.01, ∗∗∗*P* < 0.001 vs. H_2_O_2_ group.

As shown in Figure 2(a), there was no significant cytotoxicity when TMP treatment in the concentration range of 2.5 *μ*g/mL to 120 *μ*g/mL for 24 h compared to the control group. Moreover, TMP showed significant proliferative ability in the concentration range of 10 *μ*g/mL to 40 *μ*g/mL. Therefore, 10 *μ*g/mL, 20 *μ*g/mL, and 40 *μ*g/mL were selected as the optimal study concentrations.

The results of H_2_O_2_ inducing HUVEC injury showed that the treatment with 50-800 *μ*M H_2_O_2_ for 24 h reduced cell viability. Among them, 100 *μ*M H_2_O_2_ reduced the survival rate of HUVECs to about 50% (*P* < 0.001), as shown in Figure 2(b). Based on this, 100 *μ*M was selected as the modeling concentration to induce oxidative damage in HUVECs.

To evaluate the protective effect of TMP on HUVEC injury, HUVECs were exposed to H_2_O_2_ (100 *μ*M) and TMP (10, 20, and 40 *μ*g/mL) for 24 h. As shown in Figure 2(c), compared with the model group, the TMP treatment group (10, 20, and 40 *μ*g/mL) significantly increased the cell viability (*P* < 0.05).

### 3.2. TMP Promoted HUVEC Migration

**Figure 3:**
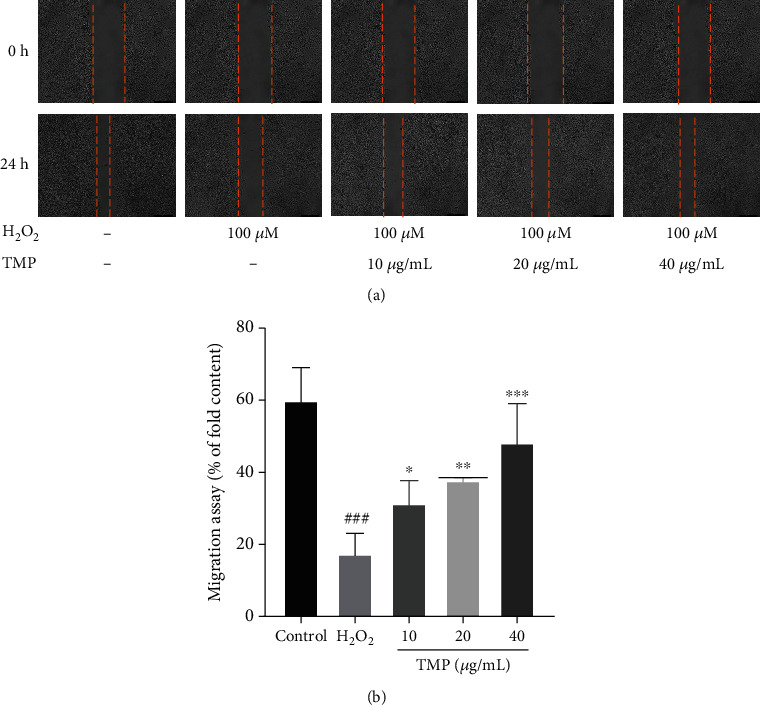
Detection of cell migration rates. (a) The effect of TMP on HUVEC migration was detected by wound healing. Photographed and observed at 0 h and 24 h time points. (b) Quantitative analysis of wound healing area. H_2_O_2_ significantly inhibited migration of HUVECs, while TMP at 10, 20, and 40 *μ*g/mL significantly promoted migration of H_2_O_2_-injured HUVECs. Values are presented as means ± S.D. (*n* = 3). #*P* < 0.05, ##*P* < 0.01, ###*P* < 0.001 vs. control group; ∗*P* < 0.05, ∗∗*P* < 0.01, ∗∗∗*P* < 0.001 vs. H_2_O_2_ group.

Endothelial cell migration is critical for angiogenesis. The effect of TMP on the migration of HUVECs was assessed by wound healing assay. As shown in Figure 3, the migration rate of the control group was 60.03%. After H_2_O_2_ (100 *μ*M) treatment for 24 h, the mobility decreased to 17.43% (*P* < 0.001), which significantly inhibited the migration of HUVECs. However, compared with the model group, the cell migration rates in the TMP groups were 31.41%, 37.85%, and 48.27%, respectively, and the migration ability was significantly increased. The experimental results revealed that TMP could promote the migration of HUVECs.

### 3.3. TMP Suppressed mROS Production Induced by H_2_O_2_

**Figure 4:**
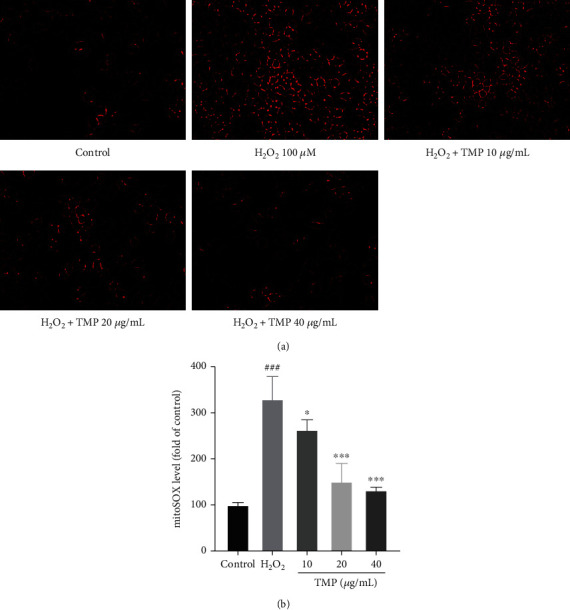
TMP inhibits oxidative damages in H_2_O_2_-stimulated in HUVECs. (a) mROS generation in HUVECs was determined by MitoSOX Red assay. (b) The mROS fluorescence intensity index was presented as the percentage of the control group. H_2_O_2_ significantly promoted mROS production of HUVECs, while TMP at 10, 20, and 40 *μ*g/mL significantly inhibited. Values are presented as means ± S.D. (*n* = 3). #*P* < 0.05, ##*P* < 0.01, ###*P* < 0.001 vs. control group; ∗*P* < 0.05, ∗∗*P* < 0.01, ∗∗∗*P* < 0.001 vs. H_2_O_2_ group.

To investigate the antioxidant effect of TMP, the mROS production was assessed by MitoSOX Red. As shown in Figure 4, after H_2_O_2_ treatment, the MitoSOX Red staining signal intensity was 285.14, which was significantly higher than that in the control group (*P* < 0.001), indicating that H_2_O_2_ can induce oxidative damage in HUVECs. While the TMP treatment groups could significantly inhibit the overproduction of mROS induced by H_2_O_2_, the signal intensity decreased to 245.25, 124.97, and 125.29, respectively. These results showed that TMP could significantly reduce mROS overproduction in HUVECs after H_2_O_2_-induced oxidative damage.

### 3.4. TMP Regulated SOD and MDA in H_2_O_2_-Indced HUVECs

**Figure 5:**
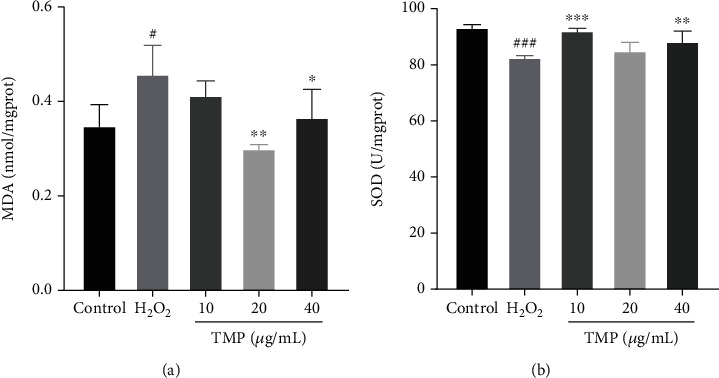
Effect of TMP on MDA and SOD activity in H_2_O_2_-injured HUVECs. (a) MDA levels of the control group, H_2_O_2_ group, and TMP groups. H_2_O_2_ significantly increased MDA levels of HUVECs, while TMP at 20 and 40 *μ*g/mL significantly inhibited. (b) SOD activity of the control group, H_2_O_2_ group, and TMP groups. H_2_O_2_ significantly inhibited SOD activity of HUVECs, while TMP at 10 and 40 *μ*g/mL significantly increased. Values are presented as means ± S.D. (*n* = 3). #*P* < 0.05, ##*P* < 0.01, ###*P* < 0.001 vs. control group; ∗*P* < 0.05, ∗∗*P* < 0.01, ∗∗∗*P* < 0.001 vs. H_2_O_2_ group.

MDA and SOD as the biomarkers of oxidative stress reflect the degree of damage to cell membrane function and integrity. As shown in Figure 5, compared with the control group, MDA was significantly increased (*P* < 0.05), and SOD activity was significantly decreased (*P* < 0.001) in the H_2_O_2_ model group, indicating that the antioxidant capacity of HUVECs was impaired. After treatment with TMP (10, 20, and 40 *μ*g/mL), the contents of MDA were significantly decreased, and the activity of SOD were significantly increased compared with the H_2_O_2_ group (*P* < 0.01 and *P* < 0.001).

### 3.5. TMP Regulates the Expression Levels of MAPK Pathway-Related Proteins

**Figure 6:**
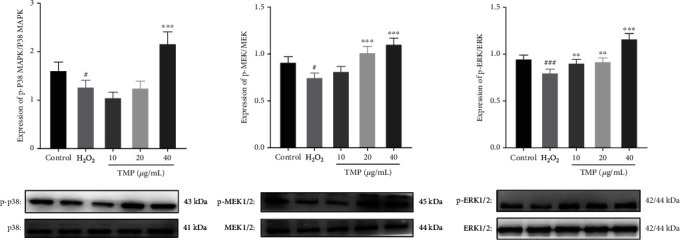
Effect of TMP on protein expression of p38, p-p38, MEK1/2, p-MEK1/2, ERK1/2, and p-ERK1/2 in HUVECs. Expression ratios of p-p38/p38, p-MEK1/2/MEK1/2, and p-ERK1/2/ERK1/2. Values are presented as means ± S.D. (*n* = 3). #*P* < 0.05, ##*P* < 0.01, ###*P* < 0.001 vs. control group; ∗*P* < 0.05, ∗∗*P* < 0.01, ∗∗∗*P* < 0.001 vs. H_2_O_2_ group.

To investigate whether TMP can protect endothelial injury through the MAPK signaling pathway, we further performed western blot assay. As shown in Figure 6, compared with the control group, the model group significantly inhibited the expression ratios of p-p38/p38 (*P* < 0.05), p-MEK1/2/MEK1/2 (*P* < 0.05), and p-ERK1/2/ERK1/2 (*P* < 0.001), while TMP treatment (10, 20, and 40 *μ*g/mL) significantly increased the expression ratios of these proteins. Therefore, these results suggested that TMP could promote angiogenesis by activating the MAPK pathway in HUVECs.

### 3.6. TMP Inhibited Apoptosis in HUVECs

**Figure 7:**
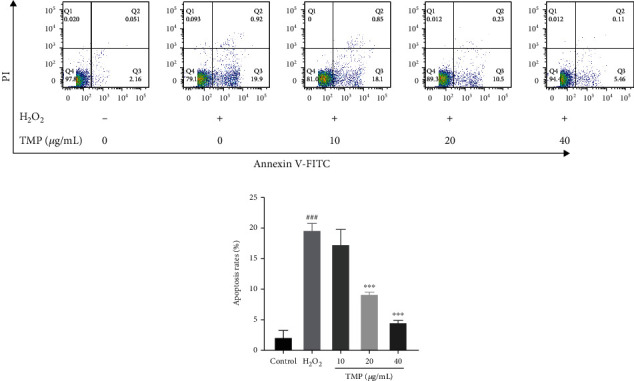
Effects of TMP on apoptosis of HUVECs. Flow cytometry detection of HUVEC apoptosis scatter plot and apoptosis rate. Values are presented as means ± S.D. (*n* = 3). #*P* < 0.05, ##*P* < 0.01, ###*P* < 0.001 vs. control group; ∗*P* < 0.05, ∗∗*P* < 0.01, ∗∗∗*P* < 0.001 vs. H_2_O_2_ group.

Inhibition of HUVEC apoptosis is an effective method to protect endothelial cell injury. Therefore, double staining with annexin V-FITC/PI was used to assess the apoptosis of HUVECs. As shown in Figure 7, H_2_O_2_ stimulation significantly promoted the apoptosis of HUVECs compared with the control group. TMP could significantly inhibit apoptosis, including early and late apoptotic cells.

### 3.7. TMP Regulated the Expression Levels of Mitochondrial Apoptosis Pathway–Related Proteins in HUVECs

**Figure 8:**
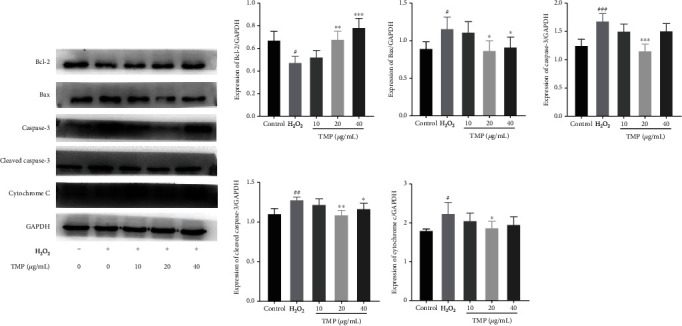
Effect of TMP on protein expression of Bcl-2, Bax, caspase-3, cleaved-caspase-3, and cytochrome c in HUVECs. Expression ratios of Bcl-2, Bax, caspase-3, cleaved-caspase-3, and cytochrome c. Values are presented as means ± S.D. (*n* = 3). #*P* < 0.05, ##*P* < 0.01, ###*P* < 0.001 vs. control group; ∗*P* < 0.05, ∗∗*P* < 0.01, ∗∗∗*P* < 0.001 vs. H_2_O_2_ group.

Mitochondria play an important role in apoptosis. To further elucidate the potential molecular mechanism of TMP inhibiting the apoptosis of HUVECs, western blotting was used to detect the changes of TMP-related apoptosis proteins in the mitochondrial apoptosis signaling pathway in HUVECs. As shown in Figure 8, TMP significantly inhibited the expression of the proapoptotic protein Bax, cytochrome c, caspase-3, and cleaved caspase-3 and promoted the expression of the antiapoptotic protein Bcl-2, thereby exerting an antiapoptotic effect.

### 3.8. TMP Restored PTK787-Induced ISV Insufficiency in Zebrafish Embryos

**Figure 9:**
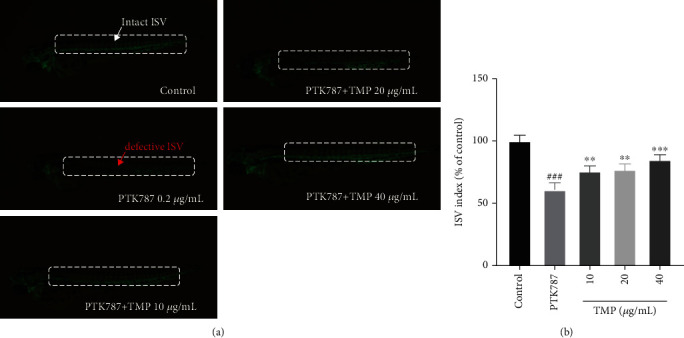
Effect of TMP on insufficient angiogenesis in Flik zebrafish embryos. (a) The 1 dpf Flik zebrafish larvae were treated with 0.1% DMSO, 0.2 *μ*g/mL PTK787, various concentrations of TMP (10, 20, and 40 *μ*g/mL) cotreated with PTK787 for 24 h. Red and white arrow indicate the defective ISV and intact ISV of zebrafish, respectively. (b) Quantitative analysis showing TMP inhibited PTK787-induced ISVs deficiency. Values are presented as means ± S.D. #*P* < 0.05, ##*P* < 0.01, ###*P* < 0.001 vs. control group; ∗*P* < 0.05, ∗∗*P* < 0.01, ∗∗∗*P* < 0.001 vs. PTK787 group.

As shown in Figure 9, 1 dpf zebrafish embryo treated with PTK787 for 24 h significantly inhibited the formation of ISVs. Therefore, we chose PTK787 to inhibit vascular structure to evaluate the proangiogenic activity of TMP in zebrafish embryos. The growth of ISVs was significantly inhibited to almost 60.87% after the treatment with 0.2 *μ*g/mL PTK787 for 24 h in AB strain zebrafish. TMP (10, 20, and 40 *μ*g/mL) treatment significantly promoted the recovery of ISVs and reversed the ISV deficiency induced by PTK787. Thus, TMP has a protective effect on PTK787-induced ISV insufficiency in zebrafish embryos.

### 3.9. TMP Decreased AH-Induced Thrombosis in Zebrafish Embryos

**Figure 10:**
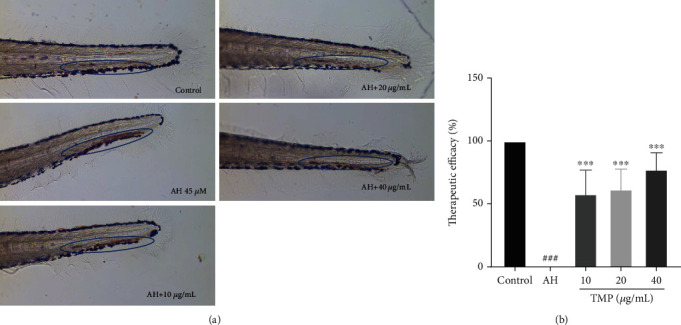
Effect of TMP on AH-induced thrombosis in zebrafish larvae. (a) The 5 dpf zebrafish larvae were treated with 0.1% DMSO, 45 *μ*M AH, various concentrations of TMP (10, 20, and 40 *μ*g/mL) co-treated with AH for 16 h. Blue circle indicates the thrombus in the caudal vein. (b) Quantitative analysis showing TMP significantly decreased AH-induced thrombosis. Values are presented as means ± S.D. (*n* = 10). #*P* < 0.05, ##*P* < 0.01, ###*P* < 0.001 vs. control group; ∗*P* < 0.05, ∗∗*P* < 0.01, ∗∗∗*P* < 0.001 vs. AH group.

To study the antithrombotic effect of TMP *in vivo*, zebrafish were stained with o-anisidine. The antithrombotic activity of TMP was analyzed by calculating the erythrocyte staining intensity of the zebrafish caudal vein. As shown in Figure 10, compared with the control group, the staining intensity of caudal vein thrombus was significantly increased in the AH group (*P* < 0.001), indicating that AH can successfully induce the zebrafish thrombus model. Interestingly, the TMP (10, 20, and 40 *μ*g/mL) treatment significantly improved caudal vein thrombosis, and the inhibition rates of thrombus were 58.09%, 61.70%, and 77.40%, respectively. These results suggest that TMP has a certain therapeutic effect on AH-induced thrombosis in zebrafish.

### 3.10. TMP Inhibited AH-Induced Oxidative Damage in Zebrafish

**Figure 11:**
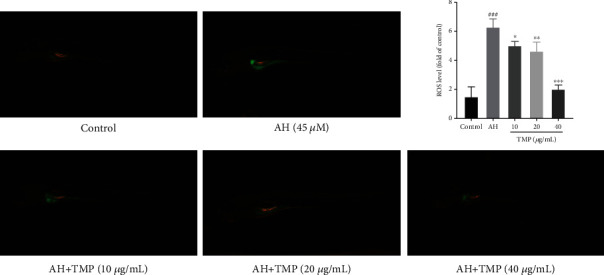
Effects of TMP on ROS levels in AH-treated zebrafish. Values are presented as means ± S.D. #*P* < 0.05, ##*P* < 0.01, ###*P* < 0.001 vs. control group; ∗*P* < 0.05, ∗∗*P* < 0.01, ∗∗∗*P* < 0.001 vs. AH group.

Oxidative stress plays an important role in thrombosis. As shown in Figure 11, AH could significantly induce the production of ROS in zebrafish. Excessive production and accumulation of ROS can induce vascular endothelial cell damage *in vivo*, eventually leading to thrombosis. However, after treatment with different concentrations of TMP, the signal intensity of DCFH-DA was significantly attenuated, indicating that TMP can improve oxidative stress in zebrafish and inhibit thrombosis through antioxidation.

### 3.11. TMP Reduced the Expression of vWF, Coagulation Factor (f7), and Fibrinogen (fga, fgb, fgg) in the Zebrafish Thrombosis Model

**Figure 12:**
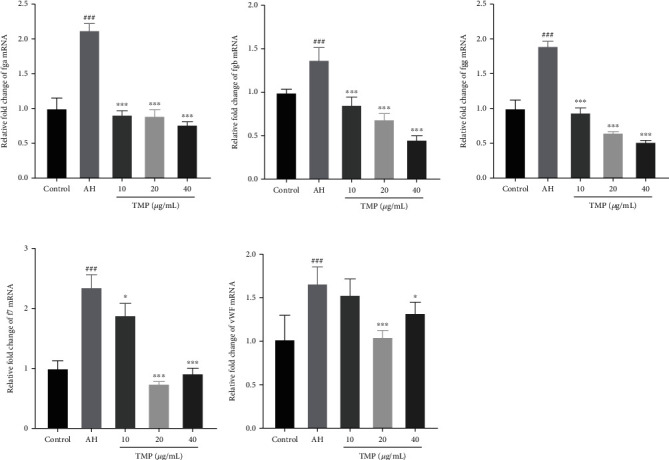
Effect of TMP on the related mRNA expression of platelet activity and coagulation cascade. TMP inhibited PTK787-induced fga, fgb, fgg, vWF, and f7 mRNA expression. Values are presented as means ± S.D. (*n* = 3). #*P* < 0.05, ##*P* < 0.01, ###*P* < 0.001 vs. control group; ∗*P* < 0.05, ∗∗*P* < 0.01, ∗∗∗*P* < 0.001 vs. AH group.

To further explore the possible antithrombotic mechanism of TMP, we quantitatively analyzed platelet activity and coagulation cascade-related mRNA by RT-qPCR. The results showed that the expression of fga, fgb, fgg, f7, and vWF mRNA was significantly upregulated after AH incubation and significantly decreased under TMP (10, 20, and 40 *μ*g/mL) treatment, as presented in Figure 12. These results suggested that the mechanism of the antithrombotic effect of TMP may be related to inhibiting the related mRNA expression of platelet activity and coagulation cascade.

## 4. Discussion

Thrombosis is a common symptom of cardiovascular diseases with high morbidity and mortality. Vascular endothelial injury, slowed blood flow, and coagulation dysfunction are important causes of thrombosis [11]. Among them, oxidative stress-induced endothelial injury, as one of the pathophysiological mechanisms that may cause thrombosis, can lead to venous wall damage. The functional integrity of endothelial cells in the vessel wall is an important protective mechanism against thrombosis [12]. It has been confirmed that endothelial injury can disrupt endothelial permeability and promote thrombosis *in vivo* [13]. Oxidative stress, apoptosis, and endothelial injury are inextricably linked to thrombosis. Once the pathological damage is induced by various stimuli such as oxidative stress in endothelial cells, the cell proliferation apoptosis and vascular damage will be affected through some signaling pathways [14]. Damaged vascular endothelial cells expose collagen, activate platelets, initiate coagulation, and gradually lead to thrombosis. Among them, oxidative stress is the initial event, and apoptosis is the core event of thrombosis. Interestingly, when ROS accumulate *in vivo*, these free radicals activate mitochondria to induce oxidative stress and then activate the MAPK signaling pathway, finally activating caspases to induce neuronal apoptosis [15]. Studies have demonstrated that the alleviation of endothelial cell oxidation and apoptosis inhibits thrombus development [16, 17]. This study found that TMP promoted endothelial cell proliferation and migration through the MAPK pathway, alleviated H_2_O_2_-induced endothelial cell oxidative damage, attenuated cell apoptosis, and protected H_2_O_2_-induced HUVECs injury. In the zebrafish model, TMP protected against insufficient angiogenesis and attenuated caudal vein thrombosis, thus having potential for antithrombotic applications.

Oxidative stress is caused by an antioxidation-prooxidation imbalance that can lead to elevated ROS and cell damage [18]. In vascular endothelial cells, ROS is a by-product of mitochondrial metabolism and mainly originates from mitochondria [19]. In the cardiovascular system, ROS plays a role in regulating proliferation, endothelial function, and angiogenesis and is one of the important factors in thrombosis [20, 21]. Previous studies confirmed the presence of high oxidative stress in thrombosis patients, suggesting that oxidative stress plays a key role in the pathological process of thrombosis [22]. Therefore, antioxidant effects can be used to prevent endothelial damage and ultimately improve thrombus formation. MDA is produced by the peroxidation of polyunsaturated fatty acids on the cell membrane, which can further cause the tissue oxidative damage and is usually used as an indicator of oxidative stress in cell membranes [23]. Studies have shown that compared with healthy controls, patients with venous thrombosis have higher levels of MDA in the body [22]. SOD is the main antioxidant in vascular endothelial cells, which can remove lipid peroxides in the body and reduce peroxidative damage to cells. Studies have found that antioxidants can protect cells from oxidative stress by increasing SOD activity, thereby preventing thrombosis [24]. High concentrations of H_2_O_2_ can be converted into oxygen free radicals and hydroxyl radicals in cells, promoting the accumulation of ROS in HUVECs to cause oxidative stress [25]. In the present study, we demonstrated that H_2_O_2_ induced oxidative stress injury in HUVECs by increasing the levels of ROS and MDA and decreasing the activity of the antioxidant enzyme SOD. However, TMP (10, 20, and 40 *μ*g/mL) treatment groups could significantly reduce the contents of ROS and MDA and increase the activity of SOD. Combined with zebrafish experiments, it was proved that TMP could significantly inhibit the production of ROS in zebrafish and inhibit thrombosis *in vivo* through antioxidation.

Elevated ROS levels are a marker of oxidative damage and induce endothelial cell apoptosis [26]. Most apoptotic signals originate from mitochondria and can be activated by a variety of stimuli including oxidative stress. Parts of the Bcl-2 family members, including proapoptotic (Bax) and antiapoptotic (Bcl-2, Bcl-xl) proteins, control mitochondrial membrane potential to regulate apoptosis signaling [27]. Among them, Bax stimulates the release of cytochrome c from mitochondria by enhancing the permeability of the mitochondrial outer membrane, thereby activating the downstream apoptosis executor caspase and initiating the apoptosis process [28]. Caspase-3, as an inactive dimer, is activated to cleaved-caspase-3 by proteolytic cleavage of a subunit, which further cleaves different substrates, leading to the expansion of the protease cascade and ultimately cell death [29]. Once vascular endothelial cells are damaged or apoptotic, the expression of phosphatidylserine will be increased, the asymmetry of membrane phospholipids will be deprived, and anticoagulant membrane components will be eliminated, thereby significantly promoting thrombosis [30]. The occurrence and regulation of apoptosis involve a variety of signaling pathways. Among them, PI3K/Akt, MAPK and Wnt signaling pathways have significant changes in thrombosis, and antiapoptotic signaling pathways can resist thrombosis by regulating endothelial cell status [14]. Therefore, modulation of apoptosis is a potential therapeutic approach for the treatment of thrombosis and cardiovascular diseases. Present studies showed that H_2_O_2_ treatment increased the expression of Bax, cytochrome c, and caspase-3 proteins and decreased the level of Bcl-2 in HUVECs, which indicated that H_2_O_2_ induced apoptosis. However, TMP reversed all these changes, suggesting that TMP has an antiapoptotic effect in endothelial cells.

Numerous studies have shown that MAPKs belong to the filaments/threonine protein kinases and play key roles in cell proliferation, migration, and angiogenesis [31]. MEK1/2 and ERK1/2 are members of the MAPK family, and once MEK1/2 is phosphorylated and activated, so will ERK1/2 [32]. Park et al. reported that rosin enhanced the migration and tube formation of HUVECs by regulating the MAPK pathway [33]. Furthermore, fucoidan has been shown to induce angiogenesis in HUVECs through the phosphorylation of MAPKs [31]. Recent studies have demonstrated that the activation of MAPKs can protect endothelial injury to resist thrombosis [34]. The results of this study demonstrated that the MAPK signaling was activated in HUVECs. Furthermore, TMP increased the phosphorylation of p38, MEK1/2, and ERK1/2, but did not affect the levels of p38, MEK1/2, and ERK1/2 proteins in H_2_O_2_-injured HUVECs. *In vivo*, a previous study showed that PTK787 can induce the deficiency in ISVs [35]. In order to study the protective effect of TMP on vascular endothelium *in vivo*, the proangiogenic effect of TMP was evaluated by PTK87-induced insufficiency of angiogenesis in Flik zebrafish, and the experimental data suggested that TMP has a proangiogenic activity. Therefore, the activation of the MAPK pathway may be the underlying cause of TMP-mediated antioxidation, antiapoptosis, and protection from thrombosis.

At present, except for the mouse thrombosis model, there are limited ideal animal models for studying thrombosis. The zebrafish model we used is different from the existing thrombosis models in that its platelets are homologous to mammalian platelets, the hemostatic mechanism is similar to that of humans, and the embryo has high transparency. It was previously reported that AH promoted platelet aggregation and ultimately induced thrombosis [36]. This study established an AH-induced zebrafish tail vein thrombosis model, and the results were consistent with previous results. The results showed that thrombosis was significantly reduced after TMP treatment. Subsequently, the possible molecular mechanism of TMP in antithrombotic was further studied. Fibrinogen is a soluble substrate for fibrin-based coagulation, including fga, fgb, and fgg, with a central role in thrombosis [37]. In addition, vWF is one of the key players in thrombosis, mediating platelet adhesion to the exposed collagen of the damaged vascular endothelium [38]. Therefore, inhibition of vWF activity would be a new preventive measure to reduce thrombotic events. Our study found that TMP could significantly reduce AH-induced overexpression of fga, fgb, fgg, f7, and vWF mRNA, suggesting that TMP may be antithrombotic by inhibiting the coagulation cascade.

However, this study also has limitations. First, although this study focused on endothelial cells and zebrafish, it is likely that the findings can be extended to other cell types and animal models, making the experiments more convincing. Secondly, future studies can verify the role of the MAPK pathway in protecting endothelial injury by using the pathway protein inhibitors. Furthermore, despite encouraging data from *in vitro* and *in vivo* studies of TMP, clinical studies of TMP are still lacking. Overall, our findings and hypotheses may at least partially reveal the potential role of TMP in the treatment of endothelial injury-related thrombosis.

To sum up, our study showed that TMP could effectively reduce the production of ROS, protect vascular endothelial injury through the MAPK pathway, and promote angiogenesis for antithrombosis. Therefore, TMP may be a potential candidate for preventing endothelial injury-induced cardiovascular diseases. These findings may provide an important scientific basis for further research on the blood-activating effect of Chuanxiong.

## 5. Conclusion

**Figure 13:**
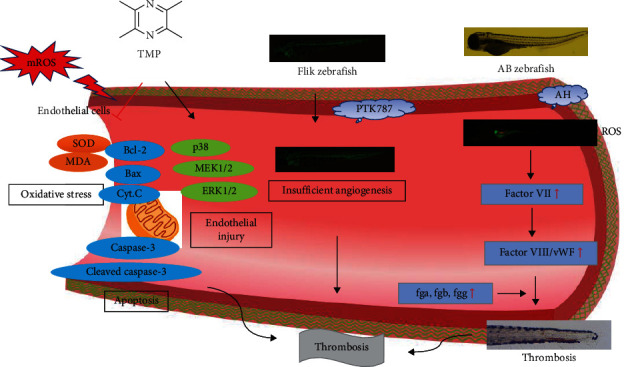
Protective endothelial injury and antithrombotic mechanisms of TMP. TMP can inhibit H_2_O_2_-induced HUVECs damage, promote the expression of MAPKs pathway proteins, inhibit oxidative stress and apoptosis, and protect endothelial cells. Meanwhile, it protects zebrafish from insufficient angiogenesis and inhibits the coagulation cascade, thereby antithrombotic.

In conclusion, our study shows that TMP has a protective effect on H_2_O_2_-induced endothelial injury and protects zebrafish from angiogenesis deficiency and thrombosis. According to molecular biological analysis, the molecular mechanism of TMP involves activating the expression of the MAPK pathway proteins, antioxidation, and antiapoptosis and inhibiting the expression of thrombosis-related genes, as shown in Figure 13. This study may provide potential new ideas for TMP to treat thrombosis caused by endothelial injury.

## Figures and Tables

**Table 1:** The gene primer sequence used for RT-qPCR.

Gene	Forward primer sequence (5′→3′)	Reverse primer sequence (5′→3′)
*fga*	5′GGCTTTGTTGGCGGAGATTG3′	5′TTGAACATCCCGCTCTGACC3′
*fgb*	5′AGAAAGTCAGCGAGGGCAAT3′	5′ATGTTCTGGGGGAAGGTGAC 3′
*fgg*	5′TCGATCATGCATGTGGTTGC3′	5′AGTAGTCTCCTCTTTGCGCTG 3′
*f7*	5′GGTGAGAAGGGTTTCTGTGGA3′	5′CCACCTCCAGATCATGCTCAC3′
*vWF*	5′GTTTACCAGTGCGTGTGCAA3′	5′AGGTGCGGGTGATGTTTTGA3′
*gapdh*	5′CGATCTGACAGTCCGTCTTGAGAA3′	5′CCATTGAAGTCAGTGGACACAACC3′

## Data Availability

The data used to support the findings of this study are available from the corresponding author upon request.

## Abbreviations

AH: Adrenalin hydrochloride
DCFH-DA: 2,7-dichlorofluorescein diacetate
Dpf: Days post fertilization
FBS: Fetal bovine serum
Flik: *Tg(fli-1: EGFP)y1* transgenic
Hpf: Hours post fertilization
H_2_O_2_: Hydrogen peroxide
HUVECs: Human umbilical vein endothelial cells
ISVs: Intersegmental vessels
mROS: Mitochondrial ROS
MDA: Malondialdehyde
MTT: 3-(4,5-dimethyl-thiazol-2-yl)-2,5-diphenyltetrazolium bromide
ROS: Reactive oxygen species
SOD: Superoxide dismutase
TMP: Tetramethylpyrazine
vWF: von Willebrand factor.
